# Self-Healing Mechanisms for 3D-Printed Polymeric Structures: From Lab to Reality

**DOI:** 10.3390/polym12071534

**Published:** 2020-07-11

**Authors:** Mohammed Dukhi Almutairi, Adrianus Indrat Aria, Vijay Kumar Thakur, Muhammad A. Khan

**Affiliations:** 1 School of Aerospace, Transport and Manufacturing, Cranfield University, Cranfield MK43 0AL, UK; m.almutairi@cranfield.ac.uk (M.D.A.); A.I.Aria@cranfield.ac.uk (A.I.A.); 2 Biorefining and Advanced Materials Research Centre, Scotland’s Rural College (SRUC), Edinburgh EH9 3JG, UK; Vijay.kumar@sruc.ac.uk

**Keywords:** 3D printing, self-healing mechanism, challenges, 3D printing structure, crack propagation, origami capsule, FDM

## Abstract

Existing self-healing mechanisms are still very far from full-scale implementation, and most published work has only demonstrated damage cure at the laboratory level. Their rheological nature makes the mechanisms for damage cure difficult to implement, as the component or structure is expected to continue performing its function. In most cases, a molecular bond level chemical reaction is required for complete healing with external stimulations such as heating, light and temperature change. Such requirements of external stimulations and reactions make the existing self-healing mechanism almost impossible to implement in 3D printed products, particularly in critical applications. In this paper, a conceptual description of the self-healing phenomenon in polymeric structures is provided. This is followed by how the concept of self-healing is motivated by the observation of nature. Next, the requirements of self-healing in modern polymeric structures and components are described. The existing self-healing mechanisms for 3D printed polymeric structures are also detailed, with a special emphasis on their working principles and advantages of the self-healing mechanism. A critical discussion on the challenges and limitations in the existing working principles is provided at the end. A novel self-healing idea is also proposed. Its ability to address current challenges is assessed in the conclusions.

## 1. Introduction

Self-healing materials, with characteristics that allow them to heal themselves when damaged by mechanical, thermal, or other causes, and to restore their original sets of properties, include metals, polymers, ceramics, and composites [1]. Self-healing can be defined as a natural process or mechanism, which enables the material to recover from the damages caused by, e.g., external phenomena through a healing process that returns the material to its original set of properties [2,3,4,5,6]. While natural mechanisms, such as the autonomous repair of bone, are lacking in traditional self-healing materials, the last decade has seen tremendous progress in the development and design of such materials [1]. Materials such as polymers and composites can exhibit characteristics of self-healing. If these materials are damaged, possibly due to fatigue, during their utilization, that damage can repair itself through self-healing mechanisms, which not only heal that material, but can also extend the working life of that material by maintaining its strength [1]. These properties are considered to be important in materials, which are more prone to damage, and where repair is not possible during the execution time.

The self-healing process in terms of materials started in the early 1970s, when the exploration of space (i.e., the Apollo mission) became a reality, and the shuttle was constructed of materials that included ammonium perchlorate-filled hydroxyl-terminated polybutadiene [7]. It was assumed at that time that materials could be damaged at the nanoscale, and that would lead to larger microscopic cracks, causing the catastrophic loss of not only materials, but also payloads. It became clear later that such damage could be self-healing.

**Figure 1:**
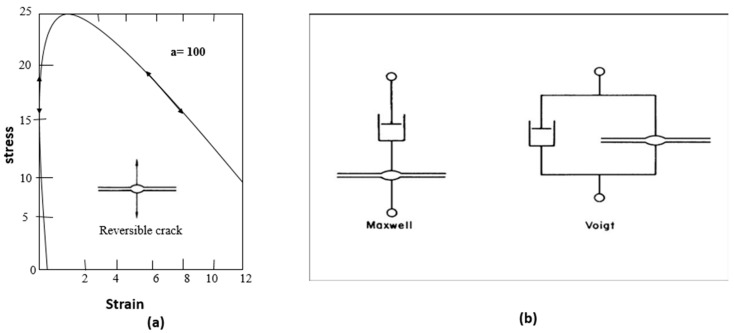
(**a**) The stress–strain response of a healing element is shown where the two parallel surfaces interact, modelled by an anharmonic non-bonded potential function. (**b**) Viscoelastic elements composing the healing element, in series (Maxwell) and in parallel (Voight). (Reproduced with permission) from [7]. Copyright: from The Royal Society of Chemistry, 2008. Figure above shows the reversible self-healing crack with basic phenomena of idealized element of self-healing, used in parallel or series with dashpots and elementary springs to the Maxwell or Voigt models, which shows yielding at critical strain.

First, damage can be measured through mechanical stress-strain, to quantify the damage and effect of self-healing methods [8]. This was known as a reversible self-healing crack, which was used later to assess the basic characteristics of self-healing elements, using, for example, the Maxwell and Voigt models [7]. Figure 1 below shows the first self-healing mechanism element, which was further improved.

Since, 1970, there have been a significant number of publications on self-healing mechanisms of different materials, especially thermoplastic and cross-linked systems. However, it was 2001 when the first article on the self-healing in polymer-based materials was published in *Nature* [7]. This triggered research into the use of self-healing polymer materials, inspired by the tremendous advantages of self-healing materials over traditional materials in engineering applications, such as electrical, automotive, biomedical, aerospace, chemical, and civil [9].

Smart polymer materials have similarities to biological self-healing phenomena, and have significantly improved the life of critical components [7,10]. The development of these kinds of systems and materials is challenging, but not impossible, and include self-healing paints and coatings, even bridges, buildings and cars [11]. Smart materials are often used in critical applications and components, to make sure the products have durability, flexibility, and are lightweight. Such materials are required to self-heal after damage. For example, presently, if a scratch is found on the paintwork of a car, it requires either expensive corrective work in a repair shop, or a significant and inconvenient repair by the owner. In such a situation, a self-healing process that ensures that the relevant material maintains its function is highly desirable, particularly if it retains its ability to reverse damage over a long time, and without delay. It would be doubly advantageous for the self-healing process to cure damage automatically without any peripheral intercession [4,5,12,13,14].

Most materials change their properties with age, and gradually decay. Some materials take longer than others; wood rots when insects or micro-organisms eat it away; however, plastic can take hundreds of years to break, or sooner with the help of fire or sunlight [9]. Some materials are very vulnerable and sensitive to breakage and sometimes very unexpectedly, such as when applied force (e.g., stress and strain) generates internal fractures. Such materials pose the hardest challenges in terms of the self-healing process [15]. With regular maintenance and inspection, it is easy to spot such problems as rotting or rusting; however, there can be cracks in crucial components where they are hard to detect, such as inside hot engines that operate at high speeds with spinning blades. There are technologies and techniques, which can be used to detect such faults, e.g., the well-known non-destructive testing technique of ultrasound scanning, that makes it relatively easy to find defects within materials or components during routine inspections. However, when the component is in use, it is almost impossible to detect a potential failure.

The mechanics of these challenges have become more complex with recent advancements in the design and manufacturing of polymer-based components [10,16]. For example, the mechanical behaviour of a component at microstructural level changes if it is manufactured using the additive method of manufacturing, rather than a conventional method [17]. In the past, self-healing mechanisms were devised for components made of conventional methods materials, but additive manufacturing (AM) offers important opportunities to include into the object means for self-repair [18].

However, polymer materials are considered to have highly self-healing and elastic properties, along with being a challenging task. A study from Zhao et al. found that with the use of 4-tris as a tetratopic linker to crosslink a poly(dimethylsiloxane) backbone,, one can obtain a self-healing polymer, with efficient outcomes upon stretching. It was also revealed that materials with strain at the break of material, such as polymers, recovered to their original length after being stretched [19]. The damaged material was also healed at room temperature—up to 93% within 1 h. Zhao et al. suggested that polymers can be used in different applications, like electronic sinks, biochips, matrixes in soft actuators, and others.

Liu et al.’s study developed epoxy Vitrimers with a combination of versatile recyclability and high performance [20]. Liu and colleagues conducted the experiment with two imine-containing hardeners synthesized from a bio-based vanillin and a petroleum-based material such as p-hydroxyl benzaldehyde. It was found that the material had good solvent resistance and cured epoxy resins, and chemical degradation products can be reused to prepare new epoxy resins. Both process and chemically recycled epoxy resins demonstrate high mechanical and retention of thermal properties, which means epoxy resin cured by petroleum-based materials.

Yuan and colleagues also designed a strategy for self-healing epoxy coatings with blending a biobased DGEDP with TPU prepolymers. The results demonstrate significant healing properties; however, healing restricted the epoxy network and chain entanglement [21]. Therefore, this study shows the rational strategy to develop a self-healing process with controlled morphology, in order to increase their functions with specific application.

Memon and Wei studied the welding and reprocessing of disulphide-containing thermoset epoxy resin, exhibiting behaviour reminiscent of a thermoplastic. The study focus on thermoset resins which is generally lack of the ability to be welded like thermoplastic because of their cross-link features. However, the increase use of fiber-reinforced composites in a large structure (e.g., rail parts, wind blades, aircraft parts, etc.) can help to better repair damages, and is compliant with environmental and recycling regulations [22].

Much research is required to develop effective self-healing mechanisms for polymer-based components made by additive manufacturing methods, such as 3D printing [16]. Furthermore, 3D printing uses a layer-by-layer approach to build a part or complete product, typically small in size, in small quantities, and with complex tailor-made geometries [23,24]. However, this layer-by-layer-based microstructure makes the mechanics of fatigue and fracture more complex than for conventionally manufactured polymer components [24]. It poses questions, such as how can we use the existing self-healing mechanisms for 3D printed components, and what new methods and materials can we possibly introduce during the printing to increase the efficiency and effectiveness of the self-healing [25,26].

In this paper, a conceptual description of the self-healing phenomenon in polymeric structures is provided, as described in the existing literature. How the concept of self-healing occurs in nature is introduced in the middle section. Later, the requirements of self-healing in current polymeric structures and components are described in detail. The existing self-healing mechanisms for 3D printed polymeric structures are also given, with a special emphasis on their working principles and advantages. A critical discussion on the challenges and limitations of the existing working principles is provided at the end. A novel self-healing idea is briefly proposed, and its possible impacts are addressed, and the challenges are highlighted in the concluding section.

## 2. Conceptual Description of Self-Healing Mechanism and Categories

Self-healing is considered to be the capability of a material to repair any damage and restore any of its characteristics that have been lost through the use of the resources contained naturally within the material, or sourced from outside [27,28,29]. A polymer with a self-healing property requires the ability to turn physical energy into a chemical and/or physical reaction, capable of repairing damage through a mechanism that is not available in conventional polymers [30,31,32]. Self-healing materials can be found with different inherent characteristics, which depend on the use to which they will be put. Self-healing can extend the lifecycle of synthetic materials and therefore has the potential for a large number of different applications [33]. Additionally, self-healing materials are capable of restoring or healing themselves without external intervention [34], and are defined as an artificial group of materials that can repair damage on one or multiple occasions to prevent the deterioration of the material, to increase its reliability and efficiency, and extend its lifespan [35,36].

**Figure 2:**
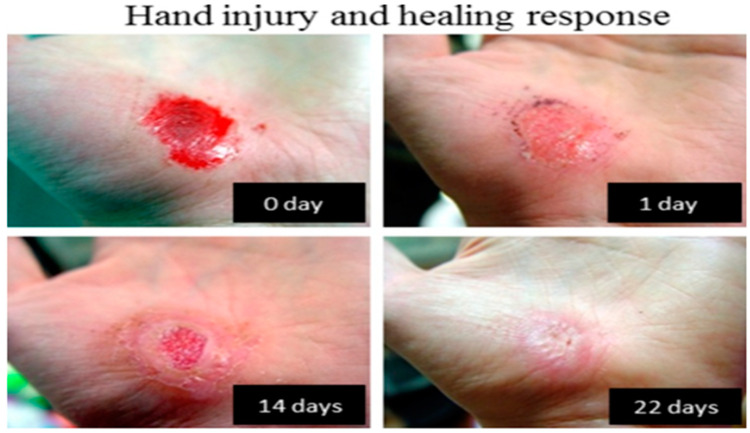
Self-healing mechanism found in nature. (Reprinted with permission from [9]. Copyright Beilstein Journal of Nanotechnology, 2018).

Figure 2 shows the self-healing mechanism in nature; the healing response of a hand injury without any external intervention. In nature, the ability to heal is an intrinsic mechanism for all multicellular organisms. This means that every organism has the potential to occupy a role in the ecosystem. Figure 2 shows the healing process in nature where the body first stops the bleeding and then the required tissue grows over the wound over a longer timescale. These are also known as sequentially controlled phases which replace the dead tissue with scar tissue and repair damaged organs [9,15].

**Figure 3:**
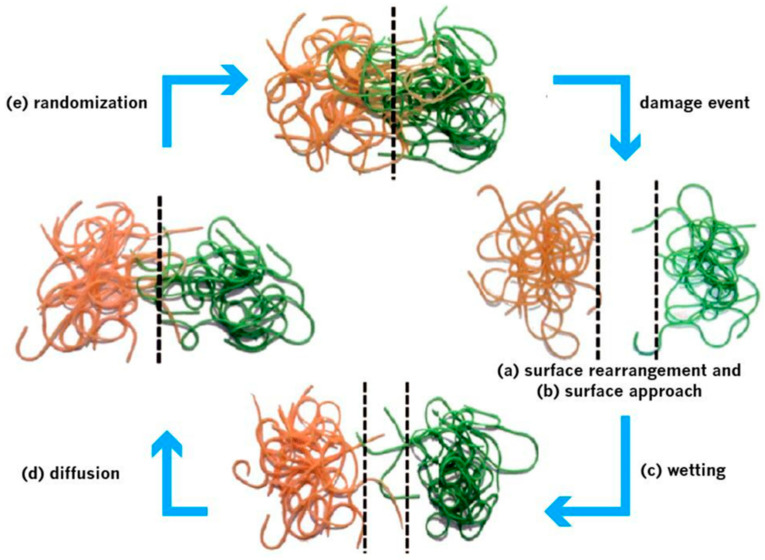
Five stages of the self-healing mechanism. (**a**) Re-arrangement of surface, (**b**) surface approach, (**c**) wetting, (**d**) diffusion, and (**e**) randomization. (Adapted from [37,39]).

Wool and O’Connor made a breakthrough in 1981, conceptualizing self-healing as a mechanism that comprises five stages: surface rearrangement, surface approach, wetting, diffusion, and randomization, shown in Figure 3 [37]. These concepts were instrumental in providing insight into the complexities involved in the recovery of ruptured polymer interfaces, and are linked with molecular inter-diffusion at temperatures above, or at, glass transition. The first stages after the occurrence of a damage event are the rearrangement of the surface and its return to original shape or condition. Healing takes place only if the broken interfaces get connected, which is why this process is considered to be essential. Crack healing rate is important as surface topography (discontinuity and roughness) caused by the crack, changes with time, temperature, and pressure. Greater surface roughness results in greater contact area, thereby resulting in higher diffusion and faster self-healing. This is an important consideration for surfaces pulled apart, due to damage that may eradicate the surface layer, and which may end the self-healing process [37]. Moreover, the surface approach stage determines whether the healing process is in point or line mode [7]. The wetting of broken surfaces by one another or by a healing agent must be ensured before the healing process can commence. This ensures that the material has a high initial chain mobility, which can also be enhanced by an increase in temperature or by the addition of solvents. These promote diffusion, resulting in the entanglement of polymer chains, thereby promoting recovery of the mechanical properties of the healed materials. This random movement due to diffusion promotes the entanglement of mobile polymer chains near the surface, and thereafter, their interpenetration into the unruptured matrix material. This stage represents an important step for the restoration of mechanical properties, and in due course, these properties are either recovered or healed [37,38,39]. Failure of the original crack interfaces can be seen during the randomization stage [7,29].

**Figure 4:**
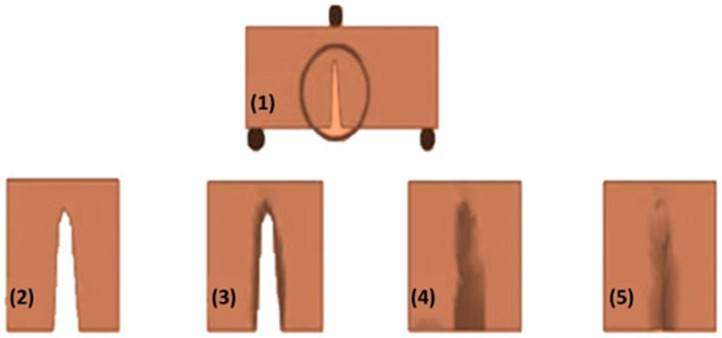
(**1**) Shows the damages to the specimen due to crack formation. (**2**) indicates how the crack can be closed by the generation of the mobile phase. (**3**) illustrates the starting of the healing action by itself or through an external source. (**4**) suggests that the mobile phase is immobilized again, and (**5**) denotes complete restoration of the original properties. (Adapted and Copyright from [40]).

**Figure 5:**
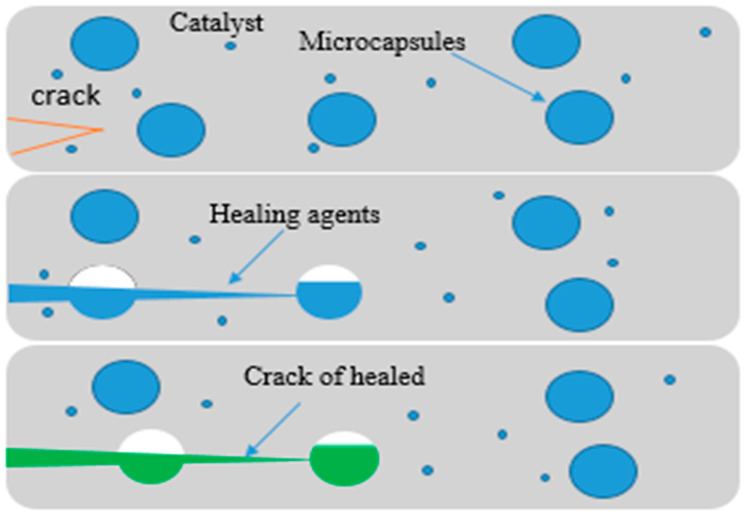
Autonomic self-healing: a micro-encapsulated healing agent is embedded with a catalyst (Grubb’s) in a structural epoxy matrix capable of polymerizing the healing fluid. (Adapted from [29]).

Self-healing mechanisms can be grouped into two categories: non-autonomic and autonomic [40]. Non-autonomic self-healing is dependent on previous information regarding the type of damage likely to be experienced, as shown in Figure 4. It requires an outside trigger (such as the presence of light, heat, mechanical, or chemical) for cracks, and damage in the polymer to be healed. The most common stimuli are heat and light, since they can be quickly applied in service environments. Conversely, the autonomic self-healing mechanism requires no previous information, as shown in Figure 5 [29]. The self-healing procedure is initiated after the damage is experienced. The inherent chemical ability is automatically released, which facilitates the process of repairing/healing any damage or rupture caused to the material. This includes encapsulation, and systems that are highly dynamic [40,41,42].

## 3. Naturally Inspired Artificial Self-Healing

**Figure 6:**
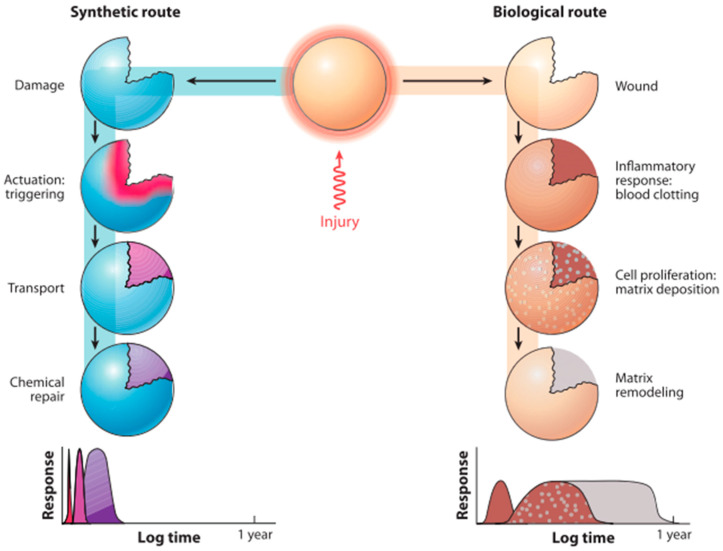
Demonstrates the different biological and artificial techniques involved in healing damaged and injuries. (Reprinted with permission from [27]).

If a human suffers a scratch or cut, a scab will develop on the wound, thus allowing the healing process to proceed with no intervention from an outside source. Based on practices observed in nature, researchers have intensively studied how self-healing abilities can be incorporated into materials fabricated from synthetic polymers, primarily to extend their operational lifetime [10,30,43,44,45]. A self-healing polymer is intended to copy the natural processes observed in animals and plants. Figure 6 depicts the artificial and biological routes to the healing of damages and wounds. In biological systems, the inflammatory response starts with blood clotting, followed by cell proliferation and matrix deposition, which may take several days, and finally the matrix remodelling, which sometimes takes several months. In the artificial system, the healing mechanism is triggered by a damage response, after which chemicals are rapidly transferred to the site of damage [7,30,44,45]. Chemical repair then occurs, extending over a period from several hours to days [27]. Any material that can automatically repair mechanical damage or do so with limited outside intervention, thus causing full or partial restoration of the material’s mechanical characteristics, can be considered as a naturally inspired self-healing material [43].

Figure 6 shows the similarity in the self-healing technique of synthetic material (in this case the insulation) and biological systems. There are three main processes: (a) activation phase, (b) transportation phase, and (c) repair phase. Thus far, the majority of investigations in the field of self-healing have specifically concentrated on polymers and polymer composites, as these materials are widely utilized in commonplace or industrial applications. Materials based on polymers that have inherent self-healing properties offer a variety of advantages, in addition to overcoming the frequently experienced issues related to polymers and their associated composite materials. For a material to be regarded as having self-healing properties, it must satisfy particular criteria. According to [29,46,47], out of the limited number of self-healing polymers, some necessitate healing agents (catalysts and monomers), while others are soft materials that necessitate a large solution (gel) or a plasticizer (rubber). Various studies have demonstrated that the lifespan of composite materials is generally constrained as a result of fatigue or other factors that cause the material to fail, due to damage experienced during use. The 3D printing of self-healing gels, using cross-linking PHEMA with EDA, to form dynamic imine bonds in the gel preparation, have been reported to have 98% recovery from mechanical damage [48]. The 3D-printing of self-healing gels remains an easy way to selectively deposit protective coatings and manufacture smart and dynamic objects [49].

The concept of the extrinsically simulated self-healing recovery of mechanical properties is also instrumental in imparting certain other functionalities; preparing smart materials with self-healing anti-corrosion coatings, self-healing electrically conductive coatings, thermo-sensitive composites, sensors, and self-lubricating and wear-resistant coatings [50]. This methodology involves the incorporation of capsules containing chemical agents with antimicrobial properties, anti-foaling properties, or biocides, with numerous types of functional coatings. Self-healing asphalt and concrete, for example, may be developed using embedded capsules, containing an asphalt binder or a concrete binder, which can provide several significant futuristic options and benefits to the construction industry. The self-healing approach can be incorporated with other methodologies like shape memory and self-sensing materials, to allow for the autonomous recovery of structural composites in extremely hostile environments [50,51,52].

## 4. Self-Healing Requirements in Polymeric Structures and Components

The existing self-healing polymers are innovative, intelligent materials that are considered to be capable of repairing themselves and reinstating their properties, with no requirement for outside sources or triggers. Usually, their healing ability relies on extrinsic healing agent encapsulations released after fractures or on intrinsic dynamic bonds, such as dynamic covalent bonds and physical bonds that develop autonomously after dissociations caused by fractures. Such polymers have been used in a wide range of applications thanks to their healing abilities, such as water membranes, lithium batteries, energy transducers, biomedical devices, flexible electronics, and soft robotics [29,46,47,52,53,54,55,56]. However, despite the success of syntheses and applications, there is still a crucial challenge due to the lack of self-healing polymers produced by 3D formation. This is seen as a bottleneck that separates artificial self-healing polymers from living materials (such as human organs), usually with practical microstructures and geometries.

**Figure 7:**
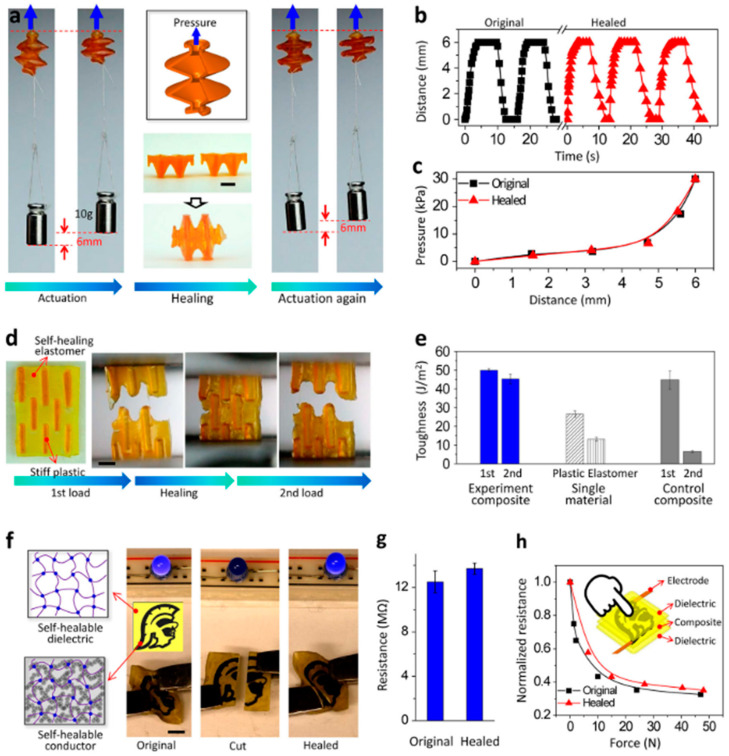
Applications of self-healing elastomers. (**a**–**c**) Self-healable 3D soft actuator. (**a**) Negative pressure actuation can enable the additively manufactured elastomer actuator to lift a 10 g weight by 6 mm. The inset shows the CAD model of the elastomer actuator. The actuator is then cut in half and brought into contact to heal for 2 h at 60 °C. The self-healed actuator can be actuated again by negative pressure, to lift the 10 g weight by 6 mm. The scale bar represents 5 mm. (**b**) The cyclic lifting distance of the 10 g weight as a function of time of the original and self-healed actuators. (**c**) The relationships between the negative pressure values and the lifting distances of the original and self-healed actuators. (**d**,**e**) Self-healable structural composite. (**d**) A notched stiff-soft composite is first uniaxially stretched until a rupture occurs, and then brought into contact to heal for 2 h at 60 °C. The healed composite is then uniaxially stretched again until a rupture. The scale bar represents 3 mm. (**e**) The toughness of the original and healed experimental composites, single materials (pure plastic and pure elastomer), and the original and healed control composites. The toughness is defined as the enveloped area of the uniaxial nominal stress-strain curves until the rupture per unit sample area. (**f**–**h**) Self-healable electronics. (**f**) A specially constructed flexible pad, 10 mm square and 1 mm thick, with a self-healable elastomer phase and a self-healable conductor phase, was used to power an LED. Once cut and healed after 4 h at 60 °C, the self-healed pad could again power the LED and sustain bending. The scale bar represents 4 mm. (**g**) The resistance of the conductive path of the pad before and after self-healing. (**h**) The relationships between the normalized resistances and the applied force of the original and self-healed force sensors. The normalized resistance is calculated as the resistance normalized by the resistance for the force-free state. The inset shows the working paradigm of the force sensor. (Reprinted with permission from [61]. Copyright NPG Asia Materials and creative commons, 2019).

The demand for an architecture of self-healing polymers is not adequately satisfied, since the current 3D methods for producing self-healing polymers only include direct writing and molding [56,57,58], which are either limited or time-consuming when developing complex 3D architectures [59,60]. This report highlights the importance of photo-polymerization-based AM for self-healing architecture such as free form. The studies also demonstrates that the rapid AM of single and more complex elastomer in multifaceted 3D geometries, in rates as fast as 12.5 mm^3^/min, produced structures that were considered to heal and restore the original mechanical strength [61]. It was shown experimentally that self-healing 3D printing elastomers with a soft actuator can restore their toughness within a range of 90% after a fracture. Such a self-healing photo-elastomer system is considered to be an efficient self-healing system mechanism and a promising source of revenue generation. The study of Yu and colleagues [61] presented molecularly designed photo-elastomer ink, which has the potential for use in stereolithography based AM, with a rapid healing mechanism. Using molecular balancing, the dual functions of self-healing and photo-polymerization were achieved using thiol-disulfide in the ink. The self-healing photo elastomer needs updating to be easily translatable to other systems, such as photopolymerization-based AM systems, that include self-propagation photopolymer waveguides, PolyJet printing, and two-photon lithography, as shown in Figure 7. Figure 7a–c presents the AM of self-healing mechanism of elastomers with different 3D architectures, and has the potential to open new methods, and is not limited to demonstrated 3D soft actuators. Figure 7d,e represents the structural composite, and Figure 7f–h represents flexible electronics, and this may also include artificial organs, bionic sensors, biomedical implants, and robotics.

The optimal means of extending the lifespan of a polymer is the self-healing mechanism, which reverses the process that ultimately causes the material to fail [43]. A perfect self-healing material is capable of sustaining its ability to sense and restore damage across the lifespan of its polymeric constituents, reinstating the material’s performance with no negative effects. It is expected that such materials will offer enhanced reliability, safety, and durability, in addition to a reduction in maintenance costs [10,43].

**Figure 8:**
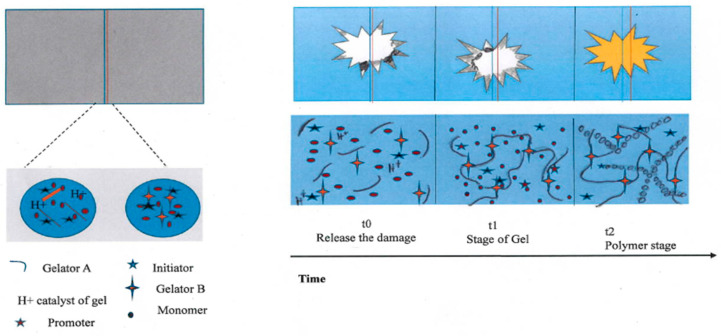
The avascular approach integrates reactive monomer solutions into a vascularized specimen. (Adapted from [62]).

In the past, studies of polymer damage restoration were with the intention replacing lost mass and recovering structural performance—the avascular approach was proposed, as shown in Figure 8. This depicts a two-stage chemistry of masks, using both rapid gelation to fill the gap and a polymer stage embracing the replacement of structural performance. At time t0, the impact damage initiates a fluid release moving towards the damage. At time t1, the gel stage occurs by covalent cross-linking. At time t2, the polymer stage (yellow) follows with the use of a two-component initiation for monomer polymerization, resulting in the recovery of structural performance [62]. In the same study, through the control of vascular delivery and reaction kinetics over 20 min, damage exceeding 35 mm in diameter was targeted, and within three hours, the mechanical function was restored. After impact damage recovery, 62% of the total energy absorbed was recovered, compared to the initial impact tests [62,63]. Renewable polymers may be possible in the future, by supplying the chemical components of the original substratum polymer, together with the flexible gel and polymer transfer.

## 5. Existing Self-Healing Mechanisms in 3D Printed Structures

### 5.1. 3D Printing of Self-Healing Materials

**Figure 9:**
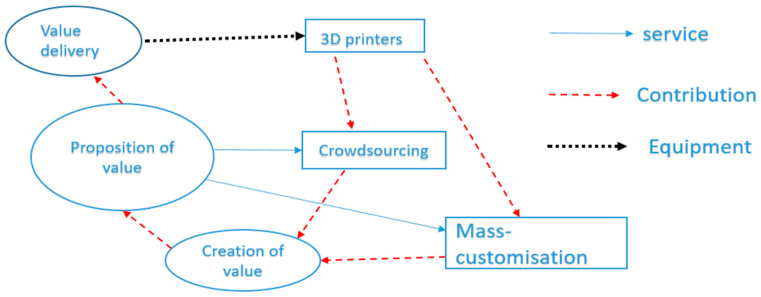
Three-dimensional printing effective interlinking between different components of the business environment. (Adapted from [66]).

The use of 3D printing has become increasingly ubiquitous across industries, as the ability to generate user-defined 3D objects with a wide diversity of materials has developed [64,65]. Evidence shows that 3D printing technologies can have a huge impact on the different sectors of business, because it can move a business from traditional processes to new innovative procedures, with a more sustainable business model [65,66]. However, sometimes, such a move can prove to be riskier as well, because there will be a significant amount of investment needed before moving toward innovative technologies or adopting the core business. Despite this fact, evidence also revealed that moving to 3D technology is considered less risky than any other innovative technologies, because it allows firms to produce products or services based on customer demands, with minimum costs, as shown in Figure 9 [66].

The self-healing process allows for the reduction of operational costs, and makes a self-healing mechanism more effective, as discussed earlier. The concept of self-healing mechanisms and 3D printing can make a significant difference and provide advantages. According to both the University of Southern California and University of Connecticut, materials with self-healing have added advantages in terms of the rapid manufacturing process using 3D printing technologies [66,67]. In 3D printing, the materials are manufactured using a photo-polymerization-based 3D printing technique, which uses a process that relies on light to solidify a liquid resin—in which it is essential to maintain the right ratio between the different chemical groups to control the materials’ characteristics. Yu and colleagues have discussed the process of the 3D printing of self-healing materials, such as 3D-printed rubber materials, which can repair damage to themselves [68].

The process of 3D printing is both AM and rapid prototyping, and involves the joining of materials to create objects based on data modelled in 3D, generally in multiple layers [69]. Moreover, 3D printing consists of various AM techniques that allow the rapid yet flexible fabrication of complex 3D structures, with different characteristics from the sub-micron to the multi-meter scale [70]. There has been extraordinary progress in the basic and applied knowledge, related to the structure and treatment of 3D printing, and its characteristics, yet many gaps remain [49]. Three-dimensional printing is an emerging technology that can be used to produce complex geometries directly, which are otherwise next to impossible to manufacture. Instead of adopting the traditional manufacturing processes [71], including molding, machining, and assembly, these steps are streamlined into a single process that greatly enhances design flexibility [72,73].

Notably, 3D printing technologies are capable of producing complicated functional or structural items through the addition of materials to reduce the crack, while also achieving adequate geometric precision [74]. Self-healing for 3D printing has attracted attention in recent years, as it opens up opportunities to build self-healing products, even with commercially available printers for individual users [75]. Self-healing products (e.g., polymers) have the capacity and capability to recover and restore functionalities, in a manner identical to the natural ability of some living creatures to regrow and repair their tissues and grow limbs [76]. This provides a window of opportunity for extending the life span and minimizing the risks and failures associated with products [75].

Healing efficiency (*n*) can be calculated using a ratio of change in the property of products and materials of interest, as shown in Equation (1):

(1) $$n=\frac{f_{healed}-f_{damaged}}{f_{virgin}-f_{damage}}$$

The *f* represents the property of interest; *f_healed_*, *f_damaged_*, and *f_virgin_* are the property of interest of the healed sample, damaged sample, and virgin sample, respectively.

While interest in self-repairing systems is rapidly growing, and despite the success of the applications of self-healing, many challenges remain for the existing synthetic self-healing materials. There are different physical and chemical approaches, which reach the advanced awareness of synthetic self-healing materials, with a significant efficiency found with biological systems. The study by Yu and colleagues have found that many complications and challenges remain in this regard, because during real life operations, a multiplicity of different applications will produce numerous different types of damages [67]. For example, if cracks are not detected at the early stages, they become hard to fix, and it is difficult to restore the material’s properties. Cracks can lead to the complete failure of the materials, and at that stage, it would be hard to intervene to make an effective repair. However, an initial or early-stage detection mechanism to fix problems could be a solution that lasts until manual or automatic intervention during a periodic inspection. The main materials known for their self-healing properties are polymers or elastomers, but there is a wide range of materials that could be used for self-healing processes, including cementitious materials, metals, and ceramics. To be practically useful, the materials must have the potential to intrinsically correct damage, help to reduce overall cost, reduce failures, and extend working life [77].

The most critical hold-up can be attributed to deficiencies in 3D shaping [57], which makes self-healing polymers distinctive from biological materials with microstructures and functional geometries [49].

**Figure 10:**
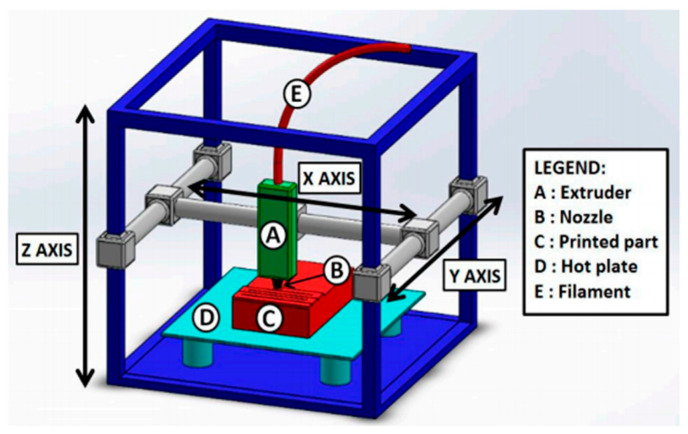
Typical fused deposition modelling (FDM) setup. (Reprinted with permission from [74]. Copyright Wiley Interscience, 2019).

Many studies have discussed different polymers and mixtures for widely used printing technologies, such as FDM, SLA, and SLS. Limitations on producing self-healing 3D printed components are mainly due to the need to understand each polymer’s characteristics and limitations [78,79]. Moreover, more control needs to be taken when setting up the printer before sending a file to the printer [80]. Besides, a dual extruder FDM, see Figure 10, may be necessarily to integrate self-healing polymers within the 3D printed components [81].

### 5.2. General Categories of Self-Healing Mechanisms Applicable to 3D Printed Structures

Self-healing mechanisms of polymer structures can be classified into two broad categories: intrinsic and extrinsic [10]. For the extrinsic approach, healing agents are sequenced through the main matrix, planted in microcapsules [29,82,83,84], or a 3D vascular network of hollow fibers [79,80] and channels [10,53,75,81], though it should be noted that the quantity and amount of transmission of the healing precursor are somewhat restricted [10]. External microcapsule layers and vascular walls are subjected to stress, when crack growth takes place and ruptures, and releases the healing agents that either react with each other or interact with the matrix to seal the crack [49]. Stiff polymer products can be incorporated, since the molecular diffusion of the matrix is not a prerequisite [65,82], and for other practical applications, extrinsic self-healing has opted to make use of hard polymer structures [76]. The correct option for a self-healing procedure will vary with nature and location of the likely damage, the chosen repair resin, the operational environment, the proximity size of the damaged zone, and the healing precursor container [75].

**Figure 11:**
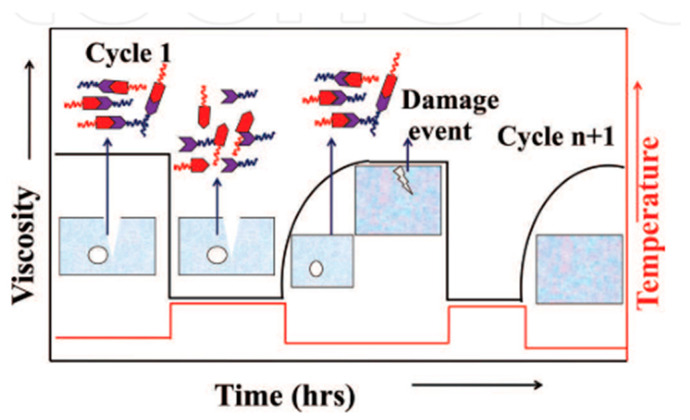
The concept of damage healing with the intrinsic approach. (Reprinted with permission from [75]. Copyright InTechopen’s, 2019).

The intrinsic approach relies solely on the inherent capacity of the materials, and comes into action through reversible covalent bonding [85,86,87] and supramolecular interactions [30,88]. To overcome the restrictions and concerns of the extrinsic approach, several intrinsic healing systems are now available. These are tailored to provide multiple healing sites during macroscopic damage [76]. Figure 11 shows the dynamic strategy of damage acceptance and management [75,84].

**Figure 12:**
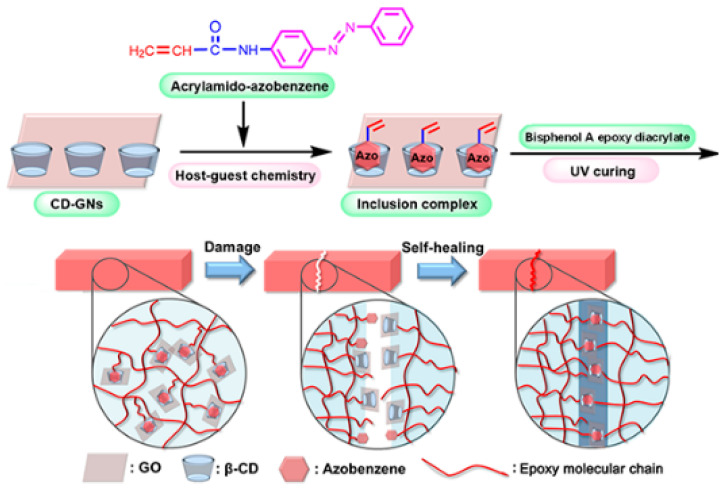
Schematic illustration of the self-healing mechanism of epoxy composite films. (Reprinted with permission from [89]. Copyright American Chemical Society, 2018).

An ongoing research study conducted on intrinsic self-healing of covalent polymers focusing on bond scission and reconnection has shown remarkable progress. The most prominent of these studies relates to the Diels–Alder (DA)-bond aided self-healing mechanism [83,85], which has been modernized and improved because of the adoption of different perspectives. Some primary works targeted varied reversible chemistries, such as the disulfide bonds, living silicon dynamic exchange of C–C bonds [86], and reversible C–ON bonds [56,88]. These studies were important in the development of intrinsic self-healing reversible covalent bond materials, thereby making available a greater number of options for specific needs, having stronger C–C bonds and multi-functional S–S bonds, as shown in Figure 12 [87,89]. In addition to these, thiolene chemistry and pH-sensitive reversible acylhydrazone bond are still to be adopted in intrinsic self-healing of damage for the restoration of mechanical properties in structural materials [88,90,91]. To provide customized solutions for practical purposes, a much greater emphasis is needed on such topics as the reproducibility of healing results, minimizing dependence on external stimuli, providing self-healing to a particular engineering material and, to ensure complete satisfaction and confidence, the preservation of the original material, with structural flexibility and integrity during crack healing.

### 5.3. Healing Mechanism for Different Material Classes

Different materials are used in 3D printing processes, including polymers, metals, and composites that have self-healing mechanisms. This means that 3D printing has an impact on how products are built, delivered, and designed. Moreover, the traditional economies of scale offered by traditional manufacturing are no longer so important.

The dependency of the required conditions, as discussed, is mainly on the nature and characteristics of materials. For metals, the desired mobility needs to be induced by increasing the temperatures to more than 800 °C. To obtain healing effectively in various types of materials, different mechanisms, each with their own strategy, are taken into consideration. As such, the encapsulation process has been adopted for various materials in a general healing approach, whereas reversible cross-link is designed for special cases related to polymers only [92,93]. Table 1 shows the mechanisms, materials and strategies for different options and classes of materials.

Comparatively less investigation has been made in terms of self-healing characteristics for metals [94]. As the healing action in metals requires mobility and higher temperatures and this poses the greater challenge of incorporating higher temperatures into the design phase, self-healing metals are categorized as nano-scale voids and macro-scale cracks [95], depending on the dimensions of the healed damage [96,97]. In contrast, concretes have an inherent ability to heal themselves, because of the distributed presence of hydrated and un-hydrated particles for the dissolution and carbonization of calcium hydroxide. These can heal smaller cracks, and restrict crack width, thereby restricting the damage at a later stage, which can also be healed. Besides, water acts as an agent for hydration and calcium hydroxide dissolution, that crystallizes when reacted with carbon dioxide. Multiple types of research has been conducted on the natural ability of such materials to self-heal. To reduce the propagation of a crack, some cementitious composites have been designed, with the addition of the reinforcement of fibers.

Polymer materials with reduced melting points or in liquid conditions are frequently utilized in the 3D printing sector as a result of their minimal weight, minimal cost, and flexible processing abilities [30]. While the geometry of 3D printed polymer objects could be significantly complex, their insufficient mechanical robustness and functionality represent considerable obstacles in terms of broader applications [98].

### 5.4. Focused Efforts Made for Developing Self-Healing in 3D Printed Structures

This section discusses, in detail, the effort made for developing self-healing in a 3D printed structure, including working principles and advantages, with visual evidence.

#### 5.4.1. Working Principle

**Figure 13:**
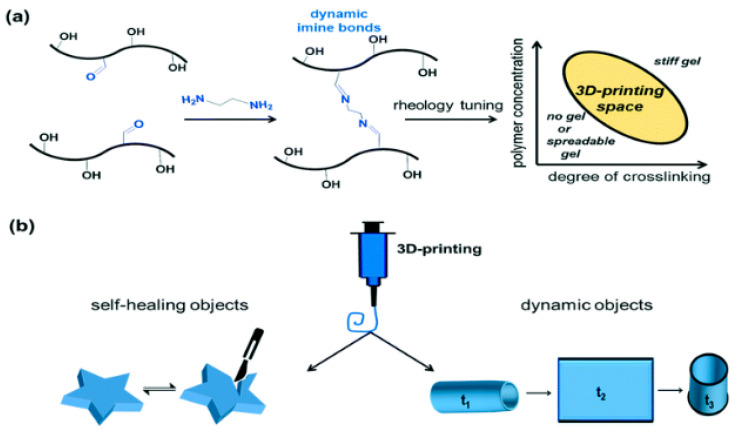
Schematic workflow for a 3D printable, self-healing working principle. (**a**) The first stage includes the synthesis and functionalization of PHEMA with pendant benzaldehyde groups, followed by a cross-linking with a diamine. The preparation of printable inks requires a delicate rheological tuning of ink formulations, and a mapping of the printability space. (**b**) After the optimization study was accomplished, selected formulations, which demonstrate a good balance between flow and self-support capabilities, were 3D-printed. The objects dis-play self-healing and dynamic properties. (Reprinted with permission from [49]. Copyright Royal Society of Chemistry, 2017).

Nadgorny and colleagues researched the 3D printing of self-healing gels, to understand recovery from mechanical damages [49]. The study presented the working principles of 3D printable, self-healing gels, as shown in Figure 13, with the following stages.

- The first step comprises the synthesis and functionalization of PHEMA (benzaldehyde-functionalized poly-2 hydroxyethyl methacrylate) with the pendant benzaldehyde group; next, cross-linking with diamine takes place. A delicate rheological tuning of ink formulations and the mapping of printable space is required to prepare printable inks [48].
- After the optimization, the study achieved good outcomes, showing a balance between self-support capabilities and the flow of 3D printed gel. Finally, the objects displayed self-healing and dynamic properties [99,100].

**Figure 14:**
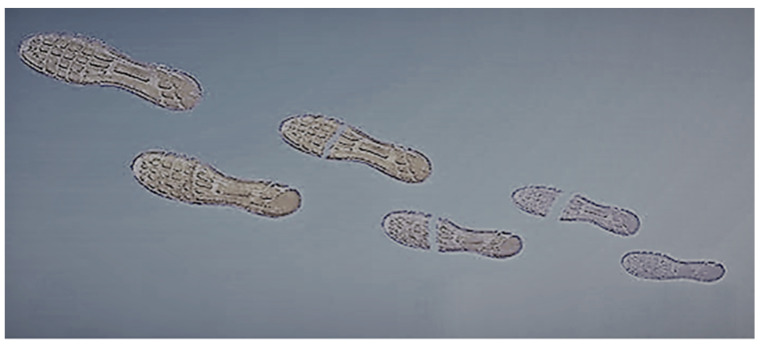
Severed 3D-printed shoe pad self-healing itself. (Adapted from [67]).

A self-healing 3D printed shoe pad was developed by Xin and Yu [63], using photo-polymerization-based 3D printing, a process that uses light to solidify the polymer into a particular shape, as shown in Figure 14.

A team from the University of Southern California experimented on additive manufacturing of the self-healing elastomers mechanism, which can be used for 3D printing, as well as 3D architecture and the self-healing of fatal fractures [61,67]. The mechanism relies on the molecular structures of photo-elastomer ink. Furthermore, the experiment used projection micro-stereolithography systems with various 3D complex geometries printed at a fast rate, e.g., 0.6 mm × 15 mm × 15 mm/min = 13.5 mm^3^/min. The main purpose was to consider how rapidly a fracture healed, and the original structure and mechanical strengths were restored. A model of the photo-elastomer in terms of the self-healing process was compared with a theoretical polymer-network-based model, and with experimental results. The self-healing materials contained both conductive and dielectric phases, and can be produced using a rapid photopolymerization capability, available with various AM systems, such as self-propagation photopolymer waveguide, PolyJet printing, stereolithographic, and two-photon lithography [101]. However, the proposed self-healing photo-elastomer system was considered a new and efficient self-healing mechanism, and suitable for 3D printing [102].

**Figure 15:**
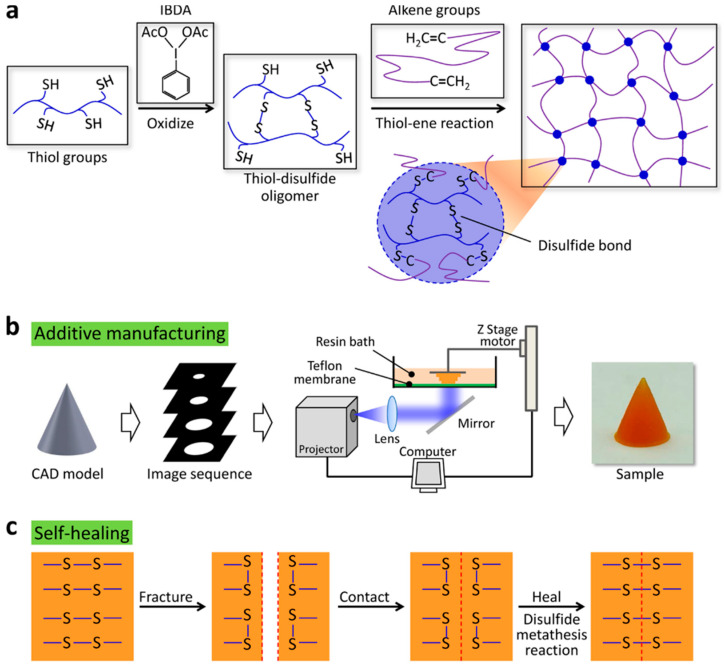
(**a**) The molecular design of the self-healing elastomer, where self-healing features and photo-polymerization were integrated with a self-healing elastomer based on the disulfide (R-S-S-R) and coexistence of thiol (R–S–H), through harnessing the high-rate and high yield thiol-ene crosslinking reaction to achieve the photo-polymerization. (**b**) the CAD model using image sequences to perform additive manufacturing to achieve efficient samples through radical initiation. (**c**) the process further goes into self-healing by harnessing dynamic disulfide bonds through reactions (disulfide) to bridge the fractured interface, as shown. The experiment was conducted with high efficient oxidant, iodobenzene diacetate (IBDA). (Reprinted with permission from [61]. Copyright NPG Asia Materials and creative commons, 2019).

The experiment used 3D-printable self-healing elastomers with a soft actuator, which can lift a weight ten times its own weight, and with the feature of restoring toughness to over 90% after fracture [103]. The materials were used without purification, and included Vinyl-terminated polydimethylsiloxanes and dimethylsiloxane (MMDS), ethanol, sudan I (photo absorber), toluene, Iodobenzene diacetate (IBDA), phenyl-bis, and phosphine oxide diacrylate (HDDA). A computer-aided design (CAD) model was used for fabricating the multi-material structure, and with an image sequence prescribed, spanning the vertical direction in the experiment, as shown in the images in Figure 15 [61].

In this experiment, the molecular design of the self-healing elastomer was one which can be used in 3D printing [102], and other applications as well.

**Figure 16:**
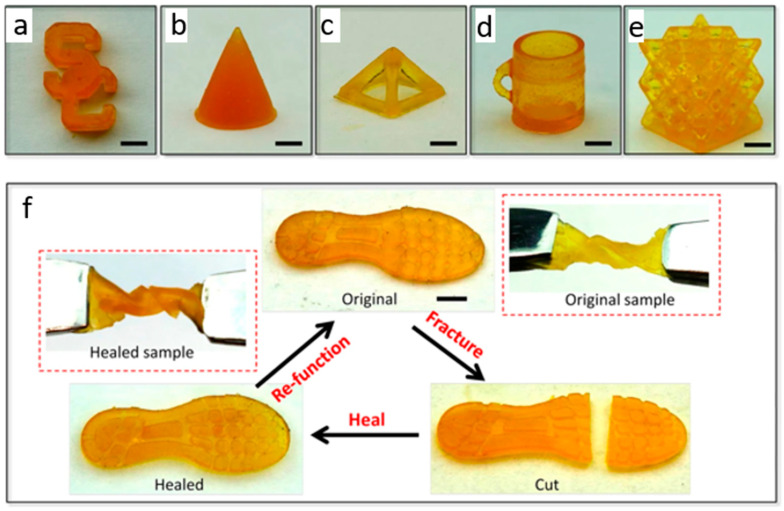
Manufacturing samples used to test self-healing mechanism with using shoe pad sample. (**a**) A logo of the South California University, (**b**) circular cone, (**c**) pyramid lattice, (**d**) cup, and (**e**) an octet truss lattice. (**f**) Self-healing of a shoes pad sample. (Reprinted with permission from [61]. Copyright NPG Asia Materials and creative commons, 2019).

An image sequence as shown above Figure 16 was sliced through the CAD model, with a fixed distance between each layer. The images were projected onto the resin bath, layer-by-layer, to give the final shape its structure. The self-healing process was enabled by a disulfide bond, and the fractured interface can be healed by the metathesis reaction of disulfide. The manufactured samples (i.e., 3D printed), circular cone, pyramid lattice, cup, and others, are shown in the figure. In terms of the self-healing process, the sample shoe pad could sustain a twist of 540 degrees. If the shoe pad was cut it, in 2 h, the healing process, at a temperature of 60 °C, returned the pad to its original shape, with properties similar to the original sample [61,104]. It was claimed that using 3D printing helped to make the product faster to manufacture, and more durable.

#### 5.4.2. Self-Healing Mechanism Advantages and Limitations

The self-healing mechanism, as obtained in 3D printed structures, has the advantages associated with 3D printing: reduced time to production, cost-effectiveness, environmentally friendly, and versatile, as almost anything that can be imagined can be produced [18]. Nevertheless, self-healing in a 3D printing structure can lead to strain release that subsequently initiates cracks that can propagate and damage the material [44,49,79,82,105,106]. However, the self-healing properties are capable of overcoming potential damage at the sub-surface level, thus allowing cracks to be healed rapidly, with no impact on the workability of the component, thereby significantly reducing vulnerability. However, because of its high customizability and capability to print complex geometries, researchers commonly use 3D-printing [23]. Additionally, a recent development in CAD software [10] and novel materials [107] further extends their 3D printing capabilities, especially for self-healing mechanisms.

There are significant limitations that exist on both the self-healing mechanism and the exploration of the study domain. Evidence shows that most of the investigations have been conducted on how could different materials can be made more damage tolerant; less research has been conducted on how materials heal the damage within the perspective of 3D printing [18]. There are many materials, such as polymers and polymer-based composite materials, that can suffer from different types of cracks, and which can lead to more severe or catastrophic damage: these are both external (e.g., environmental attacks, etc.) and internal (e.g., quality of materials, etc.) [102]. This omission is considered as a gap in the research into self-healing as applicable to 3D printing. More research needs to be conducted in this area. The limitation, in terms of self-healing for 3D printing, is cost, because more of the catalyst is required to achieve a higher degree of healing [108,109].

## 6. Challenges and Limitations (Identification of the Current Knowledge Gap)

Self-healing systems cover a wide area of interdisciplinary practice, and are carried out using various techniques. Despite the benefits of using self-healing materials, there remain challenges and limitations to the use of this mechanism. The key challenges to the self-healing mechanism is that the healing agent has a high reaction rate, which causes issues such as the incomplete curing of the epoxy [44]. Unlike previously discussed mechanisms, there are no methods presently available that can assess or evaluate self-healing systems, because measuring healing must accurately measure parameters, including thermal changes, corresponding to the impact, e.g., in milli-seconds [49]. The knowledge of the basic mechanism and the theory of damage healing remains the main challenge for the scientific community, because healing chemistries have rapid kinetics, greater stability, and greater reactivity.

The integration of the concept of self-healing into aero structures has been critical, albeit difficult, to develop, because of mechanical and thermal loadings. Sensor architecture to provide information about the healing process can be used to track components for structural health. While 3D printed sensors have several advantages, conventional sensor production methods continue to be an economical means of industrial production.

Further, much research is needed to address the challenge of transforming laboratory samples of healing mechanisms to practical applications [55]. Of the many studies on 3D-printing that have focused on concerns regarding strain release in crack initiation or damage, few have explored self-healing. Additionally, the majority of published material has shown damage cure only at the laboratory level [51,80,89], and the rheological attributes of certain structures or components mean that it is difficult to cure damage during their actual functioning [49]. The chemical reaction at the bond level is generally needed to achieve total healing, with external interruptions like heating [105]. Such reactions and requirements render it virtually impossible for the current self-healing mechanisms to be implemented, particularly in the case of printed 3D products, while they are being used in crucial applications. The vast majority of on-going research on self-healing materials will allow us to develop a new paradigm of this mechanism, and overcome most challenges, and lead to tailor-made self-healing materials for various applications. Future research needs to focus more on the practical implication of self-healing mechanisms and how effectively they could be used with 3D printing.

## 7. Novel Proposal for Self-Healing in 3D Printed Structures

The existing self-healing mechanisms are still very far from implementation, and most of the published work has only demonstrated the damage cure at laboratory level. Their rheological nature makes them complex to implement for damage cure, while the component or structure is performing its function. In most of the cases, bond level chemical reaction is required for complete healing, with external disturbances such as heating [105]. These requirements of external disturbances and reactions make the existing self-healing mechanism almost impossible to implement, especially in 3D printed products, while they are in use and working, especially in critical applications [55].

A new venture for the manufacture of smart 3D printed products could include novel origami-inspired capsules embedded within the layers of desired printed components. The capsules could provide an artificial hormone system for the 3D printed products to make their use safer and more reliable, especially in critical applications [51,80,89]. These capsules could be embedded in the form of a hormone network, while printing the desired component using conventional fused deposition modelling (FDM), and hence should be cost-effective when mass produced. This may revolutionize the self-healing ability in the structures or components, using a strain removal-based actuation for the origami-inspired capsules, in a similar fashion to the way the human hormone system actuates once a virus or bacteria enters the body. Any damage, either surface or subsurface, triggers the strain removal of the overall part/component. The capsules will start to unfold and extend themselves once a strain removal occurs at the sub-surface level in the material.

When fusing some of materials, like ABS or others, within layers, the opening may not be possible, and this might disturb the overall integrity of the structure. This is a possible potential problem. Additionally, one of the limitations of this study is using a limited source of material for capsules.

**Figure 17:**
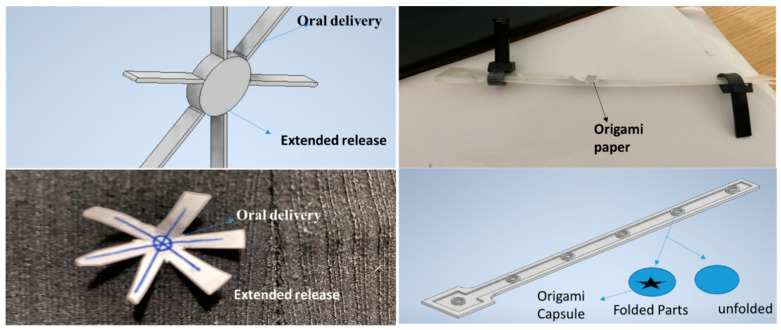
Demonstration of the concept of the proposed “origami” healing mechanism.

The basic concept of origami-inspired capsules is as shown in Figure 17. The extensions fill the damage void, while keeping the surface area covered at their original deposited location. The capsules will remain inactive in the case that the strain removal does not occur. These capsules would be designed in such a way that they are restrained within the layers of the parent components or parts, but unfold once damage or a void occurs, and overall strain energy within the material is reduced.

Origami-inspired smart capsules for artificial hormones systems will be designed and developed in future studies. Analytical and numerical approaches will be used to model the capsule’s unfolding and extension attributes against strain release [49]. The designed capsules for testing will be made using an FDM 3D printer. The mechanical design of the capsules will be tested experimentally, and verified over a range of strain release values. Re-iteration and optimization of the design will be undertaken before the capsules are embedded within the layers of a real 3D printed component. In situ characterization of mechanical strength and workability needs to be monitored and quantified during the healing process. Positive results will lead to follow-on work on the feasibility of origami-inspired smart healing for large-scale component manufacturing.

## 8. Potential Impacts of Smart Self-Healing 3D Printed Structures

### 8.1. Academic Impact

Three-dimensional printing has been incorporated into the educational curriculum, appearing in instances ranging from printed molecule models to plastic gear applications. Students can now print their 3D design models, and are thereby assisted in their learning process, allowing them to see their designs in a physical form, assisting their conceptual understanding [33]. Whilst this entire subject has been researched extensively, the challenge of synthesizing a stiff material with intrinsic self-healing capabilities remains a significant challenge. The fundamentals of damage mechanics are based on energy release during crack initiation and propagation [110,111,112,113]. The main actuation of the proposed self-healing capsules is based on strain energy release within the sub-surface.

It is important for Engineering, e.g., Civil, Mechanical, and Structural, curricula to be up-to-date and improved, to include a greater focus on building the capacity to adapt to a changing global environment [114]. Products offer a major potential for more sustainable buildings in the future. Therefore, there is a need to raise awareness that construction materials possess a huge variety of properties for beneficial use and that general-purpose approaches are dead ends from a global sustainability perspective. In developing regions, in particular, economies should take advantage of curricula that better train for the next generation of civil engineers, because new technology can be applied at a point from which major developments can emerge, so that the benefits can be readily accumulated.

### 8.2. Commercial Impact

The cost of 3D printing is going down. It is less resource-intensive, and requires less man-hours. Moreover, 3D printing allows one to have immense control, which can lead to more precise parts and low wastage. The design of a material incorporating a variety of functions remains highly complex [115]. The knowledge of smart self-healing materials is limited by the lack of work, testifying to the effect on the mechanical properties of healing-enabling constituents, such as vascular networks and microcapsules. Consequently, much research effort is necessary to resolve the various technical problems, before laboratory samples can be transformed into practice. This challenge could be overcome using unique capsule structures, or via the integration of the shape memory concept into self-healing capsules, to create a self-healing substance that offers multiple healing cycles. The implementation of such strategies will promote cost reduction and longer product life.

### 8.3. Social and Environmental Impact

Quieter/room setups of 3D printing facilities. Low Noise pollution. Filters available for control of emissions leading to low levels of environmental pollution in general [105]. The authors contend that manufacturing is a crucial factor in resolving the complicated social and environmental problems existing today, and that the overriding ethical and social challenge for producers is to assume the fundamental obligations required for effective corporate citizenship. In this vein, the various ethical and social challenges include [116]:

- The achievement of a sharing economy. This is the anticipated objective of an increasingly competitive economy, in which resources are maximized via the process in which the surplus capacity of services and goods is pooled. This refers to the economy model in which people interact within peer-to-peer (P2P) based activities for sharing access, where all have the same opportunities.
- The achievement of shared value. The objective of shared value is to generate economic value in a manner that additionally forms value for society, by focusing on its specific needs and problems, which represents a new focus for producers to determine business opportunities related to social aspects by concentrating on optimizing the competitive benefit of resolving social problems. This can be accomplished via the preconception of markets and products, the redefinition of profitability within the value chain, and the creation of clusters at the firm’s locations to support the sector.

## 9. Conclusions and Future Perspectives

Examples of autonomic and non-autonomic self-healing materials, where the material with a healing mechanism automatically reacts to damage, have been successfully demonstrated. Self-healing polymers can be considered as a new class of smart materials, which have the feature and characteristic of an extended lifetime by repairing itself when damaged, without any external intervention, and can take different forms, that can be categorized as either intrinsic or extrinsic. These polymers possess the ability to heal different kinds of damage or fractures, and while considerable progress has been achieved recently in the area of 3D printing of polymers, this has to be extended to include self-healing polymers.

At the core of this suggested development is the understanding that gradual enhancements will be unable to satisfy the challenging demands of the future global environment, and what is required is a comprehensive step-change that involves the different academic, social, and commercial challenges to be identified. However, man-made self-healing materials pose substantial challenges by requiring complex structures. Although a large number of papers on the self-healing mechanism are published every year, the study of self-healing concepts remains an active field. Within the context of self-healing materials, many areas have been explored to achieve a common goal to prolong the functional life of composite structural elements, while reducing costs. Interest in the areas of 3D printing self-healing development, including new improvements in technology, has increased in the last few years.

The promising contribution in the field of damage mechanics is the development of smart capsule dynamics at the micro-level, which can allow the added material to unfold in the same direction when the strain is removed, due to crack initiation or propagation. No external intervention or initiation is required in the proposed self-healing system. In academic terms, this has importance for the currently published methods, and will, therefore, contribute to the advancement of knowledge. Computer simulations are also an open window of opportunities to design innovative self-healing nano-systems, and provide effective direction and guidance to the efforts of researchers and scientists for the fabrication of self-repairing systems.

## Figures and Tables

**Table 1:** Healing mechanism for different materials; the symbol indicates the expected or demonstrated level of success (tick for applicable and cross for not applicable).

Mechanisms	Type of Materials		Name of Materials
	Polymers & Polymer Composites	Metals	
Encapsulation	✓		Polycarbonates, cement, ABS, poly(urea-formaldehyde) and polystyrene
Increase of temperature	✓	✓	Glass, aluminium
Expanding phases	✓	✓	powder or slurry
Separation phases	✓		Ionomers, epoxy
Reversible crosslinks	✓	×	Diels-Alder reactions, polycyclopendiene
Channel transport	✓		PLA (Polylactic Acid)
Biological processes		×	Polyurethane (PU)
Electro-chemical process	×	✓	Polycarbonates, polyethylene, plastic
